# Regulation of *cel* Genes of *C. cellulolyticum*: Identification of GlyR2, a Transcriptional Regulator Regulating *cel5D* Gene Expression

**DOI:** 10.1371/journal.pone.0044708

**Published:** 2013-01-22

**Authors:** Imen Fendri, Laetitia Abdou, Valentine Trotter, Luc Dedieu, Hédia Maamar, Nigel P. Minton, Chantal Tardif

**Affiliations:** 1 Laboratoire de Chimie Bactérienne, CNRS UMR 7283, IMM, Marseille, France; 2 Aix Marseille Université, Marseille, France; 3 Clostridia Research Group, Centre for Biomolecular Sciences, School of Molecular Medical Sciences, University of Nottingham, Nottingham, United Kingdom; University of Kansas Medical Center, United States of America

## Abstract

Transcription and expression regulation of some individual *cel* genes (*cel5A*, *cel5I*, *cel5D* and *cel44O*) of *Clostridium cellulolyticum* were investigated. Unlike the *cip-cel* operon, these genes are transcribed as monocistronic units of transcription, except *cel5D*. The location of the transcription initiation sites was determined using RT-PCR and the mRNA 5′-end extremities were detected using primer extension experiments. Similarly to the *cip-cel* operon, *cel5A* and *cel5I* expressions are regulated by a carbon catabolite repression mechanism, whereas *cel44O* and *cel5D* expressions do not seem to be submitted to this regulation. The role of the putative transcriptional regulator GlyR2 in the regulation of *cel5D* expression was investigated. The recombinant protein GlyR2 was produced and was shown to bind *in vitro* to the *cel5D* and *glyR2* promoter regions, suggesting that besides regulating its own expression, GlyR2 may regulate *cel5D* expression. To test this hypothesis *in vivo*, an insertional *glyR2* mutant was generated and the effect of this disruption on *cel5D* expression was evaluated. Levels of *cel5D* mRNAs in the mutant were 16 fold lower than that of the wild-type strain suggesting that GlyR2 acts as an activator of *cel5D* expression.

## Introduction

*Clostridium cellulolyticum*, an anaerobic mesophilic bacterium produces numerous enzymes that efficiently degrade cellulose and hemicelluloses, the major polymers of plant cell walls [1]. Most of the known enzymes are assembled into high molecular mass enzymatic complexes named cellulosomes [1], [2]. These extracellular complexes act very efficiently on crystalline cellulose [3], liberating soluble oligosaccharides which are used by the bacteria as carbon and energy sources [4]. Each complex is composed of up to eight enzymatic units bound to one scaffoldin (CipC) [5], [6]. This scaffoldin contains cohesin modules that serve as anchoring points for the enzymes via a strong interaction with enzyme-born dockerin modules [7]. Cellulosomes produced by *C. cellulolyticum* grown on cellulose contain at least thirty six dockerin-containing proteins [8]. The majority of these proteins are Glycoside Hydrolases belonging to families 5, 8, 9, 10, 11, 18, 26, 27, 44, 48 and 74 of the CAZy database (http://www.cazy.org) [8]. Sixty two ORFs that potentially encode dockerin-containing proteins were recently found in the genome sequence of *Clostridium cellulolyticum* strain ATCC 35319 (http://www.ncbi.nlm.nih.gov; GI: 220927459). All these genes are largely distributed on the chromosome, except two large clusters. The *cip-cel* cluster begins with the gene encoding the scaffolding protein CipC [9] followed by the gene encoding the major cellulosomal cellulase Cel48F and nine other dockerin-bearing enzymes. The *xyl-doc* cluster encodes 14 cellulosomal hemicellulases [10]. How the organism regulates the expression of such large number of genes for biomass degradation remains a standing question. It is also unknown how the bacteria sense the presence in their environment of plant cell wall polymers that are typically insoluble substrates [11] and incapable of diffusing into the cell, and what signaling mechanisms it uses to regulate gene expression accordingly.

The cellulases encoded by the *cip-cel* cluster are essential for the building of efficient cellulose-degrading cellulosomes [12].Transcriptional analysis of the *cip-cel* cluster showed that it was an operon with a primary transcript processed in varying-size transcripts. The most stable secondary transcripts carry *cipC*, *cel48F* and *cel9E* [13]. Messenger processing thus appears to participate in the regulation of cellulolysis gene expression. The operon promoter is located at around – 650 bases upstream the *cipC* start codon and its activity is strongly influenced by surrounding sequences [13], [14]. These variations may be due to the sequence-dependent conformation of the region and/or to the binding of a combination of “regulators” upstream and/or downstream from the promoter site. A catabolite-responsive element (CRE) located 414 nucleotides downstream from the transcriptional start site was found to be involved in a carbon catabolite repression mechanism of regulation [14].

It is still unknown if the expression of the various individual *cel* genes is subject to any type of regulation. Among the well known *cel* genes from *C. cellulolyticum*, *cel5A* and *cel5D* encode cellulosomal family 5 endocellulases which exhibit similar substrate specificities [15], [16]. *cel5I* [17] encodes another family 5 cellulase which do not bear any dockerin-module; its three C-terminal S-Layer Homolog sequences highly suggest a cell surface location. The latter gene is preceded by another *cel* gene but in the opposite direction: *cel44O* [18], which codes for a cellulosomal protein. Here we characterize the transcripts of these genes and measure their expression levels in cellulose based media. Using promoter transcriptional fusions, we show that the *cel44O* promoter is stronger than those of *cel5A*, *cel5D* and *cel5I* and that the four genes appear to be subjected to diverse regulations: *cel5A* and *cel5I* are submitted to carbon catabolite repression, whereas *cel44O* does not. GlyR2, a novel transcriptional regulator belonging to the AraC/XylS family encoded by a gene upstream of *cel5D* [18] is shown to be involved in the regulation of *cel5D*.

## Materials and Methods

### Bacterial strains, plasmids and culture conditions

*Escherichia coli* DH5α and BL21 (DE3) Rosetta pLysS were used as the host strain for routine cloning and for GlyR2 production, respectively. Luria-Bertani (LB) broth, Turbo broth and LB agar were used for *E. coli* liquid and solid cultures, respectively. *C. cellulolyticum* ATCC 35319 and derivative strains were grown anaerobically at 32°C in basal medium (BM) or minimal medium (MM) supplemented with cellobiose (2 g liter^−1^; Sigma), MN300 cellulose (5 g liter^−1^; Serva), or MN300 cellulose (5 g liter^−1^) plus cellobiose at a concentration of 4 g liter^−1^ [3]. Colonies of *C. cellulolyticum* were isolated on solid medium (basal medium supplemented with 8 g liter^−1^ agar). pGEM-T Easy (Promega) was used as the PCR cloning vector in *E. coli* and pPSV as shuttle vector for the quantification of promoter activity in *C. cellulolyticum* [14]. pET22b+ was used as overproducing vector for GlyR2 production in *E. coli*. pMTL007 was used as described by John T. Heap *et al* for gene inactivation in *C. cellulolyticum* [19]. Competent cells of *C. cellulolyticum* were prepared and electrotransformed as previously described [20]. The concentrations of the antibiotics used for selection were as follows: ampicillin, 100 µg ml^−1^ and chloramphenicol, 34 µg ml^−1^ for *E. coli*; erythromycin, 10 µg ml−^1^ and thiamphenicol, 2.5 µg ml^−1^ for *C. cellulolyticum*.

### RNA isolation

Total RNAs were isolated from cells grown on cellulose-containing BM (800 ml). The cells were collected at the end of the exponential phase of growth (6 days) by pipetting, taking care to not disturb the sedimented cellulose. After centrifugation, cells were resuspended in 2.5 ml of lysis buffer (30 mM Tris-HCl, pH 8, 100 mM NaCl, 5 mM EDTA, 1% SDS) and RNAs were purified as previously described [13], [21]. Total RNAs were quantified by spectrophotometric analysis at 260 nm using a Nanodrop 2000C apparatus (Thermo Scientific). PCRs were performed to check the absence of DNA.

### Northern blot analysis

RNAs were denatured in RNA sample buffer (50% formamide, 40 mM MOPS [morpholinopropanesulfonic acid; pH 7], 10 mM sodium acetate, 1 mM EDTA, 2.2 M formaldehyde, 8.33% glycerol) at 65°C for 5 min and separated by electrophoresis through 0.8% agarose gel containing formaldehyde (0.22 M) in running buffer (40 mM MOPS [pH 7], 10 mM sodium acetate, 1 mM EDTA, 0.22 M formaldehyde). RNAs were transferred overnight to a positively charged nylon membrane (Roche Applied Science) by capillary transfer using 20× SSC buffer (1.5 M NaCl, 0.15 M sodium citrate [pH 7]; Promega), and hybridized with an excess of α ^32^P-labeled antisense RNA probes in the Ultra-Hyb hybridization solution (Ambion) at 68°C overnight [13]. Hyperfilm (Amersham Biosciences) were used for autoradiography. The different antisense RNA probes were synthesized from the linearized appropriate constructs [pSPT18celA, pSPT18celI (pSPT18 derivatives carrying a 1-kb fragment of *cel5A* and *cel5I*, respectively), pGEM-TcelO and pGEM-TcelD (pGEM-T Easy derivatives carrying a 1-kb fragment of *cel44O* and *cel5D*, respectively)] by *in vitro* runoff transcription using the SP6 or the T7 RNA polymerase (Roche Applied Science) as previously described [13].

### RT-PCR

Analytic RT-PCRs were performed from 100 ng of total RNA with the Titan One Tube RT-PCR kit (Roche Applied Science) as previously described [13]. Products were displayed on a 2% agarose gel and visualized by staining with ethidium bromide.

For quantitative RT-PCRs, 500 ng to 1 µg of total RNAs were reverse transcribed using Superscript III (Invitrogen) and 100 ng of random primers according to the manufacturer procedure. The cDNAs were mixed along with 150 nmoles of primers (Table 1) and were then subjected to qRT-PCR using an Eppendorf Mastercycler ep *realplex* and SYBR Premix Ex Taq, according to the manufacturer specifications (Takara). The *rpoD* gene target was used as an internal control. Data analysis and normalization were performed with the software supplied with the Mastercycler.

**Table 1:** Primers used in the study.

**For construction of the His6-tagged GlyR2 protein**	
His6-GlyR2 Forward	CCCTATACATATGATTAATGACAATCAATCAATATTTAAT
His6-GlyR2 Reverse	GTGCTCGAGCAATTTGTATTTTGCTTTTGTGAAACCG
**For ** ***catP*** ** gene reporter transcriptional fusions**	
PrA Forward	TTAGTAAATCTTAAGCCAATAAAGC
PrA Reverse	TAATAATAATAGTGGTAGCAGTAGAA
PrD Forward	GGGATACAAGTAAGAGATTTAA
PrD Reverse	GTATTAATTTTACATATTATCTAAT
PrO Forward/PrI Reverse	GTATAAGGTTAAATAATTGGAAATCA
PrO Reverse./PrI Forward	TTTATGTTTTTTACTCATTCGCTGG
**For RT- PCR**	
AD1	GTAATTCTTTGGATAATCGG
AD2	GTAATTAACTGTAAAATTTGCAATT
AD3	CGAGGTCATTGGTACATCC
AR1	CCCTTGATTAATAATAATAGTGG
DD1	AATATGTAAAATTAATACAGGGG
DR1	AAGTGTATGCTGATCCCAACG
DD2	GTCATTAATCATACTATCTTCC
DR2	AATAATACTGCACGATATAATCAG
DD3	CGTTGATGCAATAAACCTC
GlyR1	CATTTGTAATCCAATAAGGGGAAG
GlyD1	AATTAGTACTATTTAAATCCTAGTAAGG
GlyD2	CATAGCACTCAATTGTAATATATTACTC
GlyD3	CCCTGTATTAATTTTACATATTATC
OR1	ATCAGTTATTACTACACCCGGTTC
OD1	GAATTAATTATCATTTGCCAACATTGG
OD2	TCAAATTTTTATAAATCGCCC
OR2	TCATTCGCTGGCATTTGAGATAAA
OD3	GTTTTATGGACAATTGATTTATGGTG
OD4	CCATTCTGATACTAAGGCAATCAATAC
ID1	CATATTAAATTGTAAATCAATCCTAC
ID2	GCATACATTATATATACAGTTCTCC
ID3	CACCATAAATCAATTGTCCATAAAAC
IR1	GAATTCTTTTATTGATGTTTCTA
**For qRT-PCR**	
5’qtcel5D	TTCGAAGGTGCTATGCAGTG
3’qtcel5D	GCCATTTCTCCCAATCTTGA
5’qtrpoD	TGGATGCCTTTGAGGAAATC
3’qtrpoD	TTAAAAGGGGGACCTTACCG
**For construction of ** ***GlyR2*** ** mutant**	
GlyR2 IBS	AAAAAAGCTTATAATTATCCTTAATTATCCTTTTGGTGCGCCCAGATAGGGTG
GlyR2 EBS1d	CAGATTGTACAAATGTGGTGATAACAGATAAGTCCTTTTGAATAACTTACCTTTCTTTGT
GlyR2 EBS2	TGAACGCAAGTTTCTAATTTCGATTATAATTCGATAGAGGAAAGTGTCT
EBS univ	CGAAATTAGAAACTTGCGTTCAGTAAAC
**For primer extension**	
DR2	AATAATACTGCACGATATAATCAG
GlyR2	CAATTTCTAGTATTAATATAATGATAACAC
OR2	TCATTCGCTGGCATTTGAGATAAA
IR2	TATTGCATCACTTATATTTCGCTGT
AR2	CATAAAGTGCTGTATTTCTTAC
**For probe synthesis (EMSA)**	
GlyR1	CATTTGTAATCCAATAAGGGGAAG
DR3	GGCGTCGAACTGGAAAGTG
GlyR2	CAATTTCTAGTATTAATATAATGATAACAC
DR1	AAGTGTATGCTGATCCCAACG

### Primer extension

Total RNA was reverse transcribed using the Superscript III reverse transcriptase (Invitrogen) and a radioactive 5′-end-labeled primer as previously described [13]. Extension products were analyzed on a 6% polyacrylamide sequencing gel. To map the exact transcriptional start site, sequencing reaction mixtures were used as ladders. The sequencing reactions were performed on recombinant plasmids containing the *C. cellulolyticum* DNA region analyzed by primer extension. The Thermo Sequenase Cycle Sequencing kit (USB) was used for all sequencing reactions according to the supplier's protocol.

### GlyR2 cloning and purification

To construct a His-tagged version of the GlyR2 protein, the coding region was amplified by PCR from *C. cellulolyticum* genomic DNA using the primers His6-GlyR2 Forward and His6-GlyR2 Reverse (Table 1) which incorporated the NdeI and XhoI restriction sites, respectively. The amplicon was digested with the appropriated enzymes and ligated with NdeI/XhoI-digested pET22b+. *E. coli* BL21 (DE3) Rosetta pLysS cells were transformed with the resulting plasmid named pET*glyR2*. The recombinant strain was grown at 37°C in Terrific Broth with ampicillin and chloramphenicol up to OD_600_ = 0.5 before induction by adding 50 µM IPTG and further incubation during 16 h at 17°C. The harvested cells were disrupted with a French press. The soluble extract in 30 mM Tris-HCl buffer (pH 8) was loaded onto a Nickel-nitrilotriacetic acid (Ni-NTA) column (Qiagen). His-tagged proteins were eluted with 30 mM Tris-HCl (pH 8) 250 mM imidazole (Fluka) buffer. The eluted fraction was dialyzed against 30 mM KH_2_PO_4_ buffer (pH 6.5) and concentrated using a microconcentator (10-kDa cutoff), (Vivaspin, Vivasciences) before loading onto the cation exchange chromatography-S Sepharose Fast Flow column (GE Health Care Life Sciences) equilibrated with the same buffer. The protein was eluted with a step gradient of NaCl and was found in the fraction containing 1 M NaCl. The 30 mM Tris-HCl buffer (pH 8) dialyzed protein was analyzed by SDS-PAGE for purity estimation. GlyR2 represented ∼20% of the enriched fraction. The protein concentration was estimated by the method of Lowry [22] at 1 g.L^−1^ (∼200 ng.L^−1^ of GlyR2).

### Electrophoretic Mobility Shift Assays (EMSA)

The DNA fragments containing promoters *glyR2* and *cel5D* were amplified by PCR using GlyR2/DR1 and GlyR1/DR3 primers, respectively (Table 1), purified on 1% agarose gel and 3′-end labeled with biotin using a Biotin 3′-End DNA Labeling Kit (PIERCE). All EMSA were performed on 6% polyacrylamide gels in 30 mM Tris-glycin (pH 8.3), 1 mM EDTA buffer. Each EMSA reaction mixture contained 10 µg of sonicated herring sperm DNA, 1× LightShift EMSA kit binding buffer (Pierce), 1× LightShift loading dye (Pierce), and appropriate amounts of the DNA probe and the protein preparation. For competitive inhibition of the binding reaction, 100× of unlabeled fragment was added to the mixture. EMSA gels were electro-blotted onto Whatman Biometra Fastblot. Signal development followed the LightShift Chemiluminescent EMSA kit protocol (Pierce) with BioMax films (Kodak) for luminescence detection.

### Construction of catP transcriptional fusion and chloramphenicol acetyl-transferase (CAT) assays

The *cel5A*, *cel5D*, *cel5I* and *cel44O* promoter regions (−653 to −20, −797 to −15, −854 to −16, −856 to −18/ATG, respectively), were amplified by PCR from *C. cellulolyticum* genomic DNA using the primer pairs PrA Forward/PrA Reverse, PrD Forward/PrD Reverse, PrI Forward/PrI Reverse and PrO Forward/PrO Reverse, respectively (Table 1). The amplicons were inserted into the pGEM-T Easy vector. The pPSVcelA, pPSVcelD, pPSVcelI and pPSVcelO vectors containing transcriptional fusions were constructed by ligating the SphI-SacI fragments from recombinant pGEM-T Easy vectors with the pPSV promoter probe vector digested with the same enzymes. The pPSV derivatives were transferred to *C. cellulolyticum* by electrotransformation. Transformants were isolated on selective solid medium containing 10 µg mL^−1^ as previously described [14]. CAT activities, expressed in nanomoles per minute per mg of protein, were measured as previously described [14].

### Mutant construction

*C. cellulolyticum glyR2* was constructed as described by Heap et al [19], using a mutagenesis system based on the mobile group II intron from the *ltrB* gene of *Lactococcus lactis* (Ll.ltrB) adapted to function in clostridial hosts. The intron sequence was adapted to the target by replacing part of the intron carried by the pMTL007 shuttle plasmid by a PCR fragment. Primers used (Table 1) for the synthesis of the PCR fragments were designed using the TargeTron Design Site (sigma-aldrich.com/targetronaccess). Competent cells of *C. cellulolyticum* were prepared and electrotransformed as previously described [20]. Transformant selection was based on thiamphenicol resistance. Integrants were selected on erythromycin-containing medium after overnight induction with 3 mM IPTG. The absence of *glyR2* transcript in the mutant strain was confirmed using quantitative RT-PCR.

## Results

### cel5A transcription

**Figure 1:**
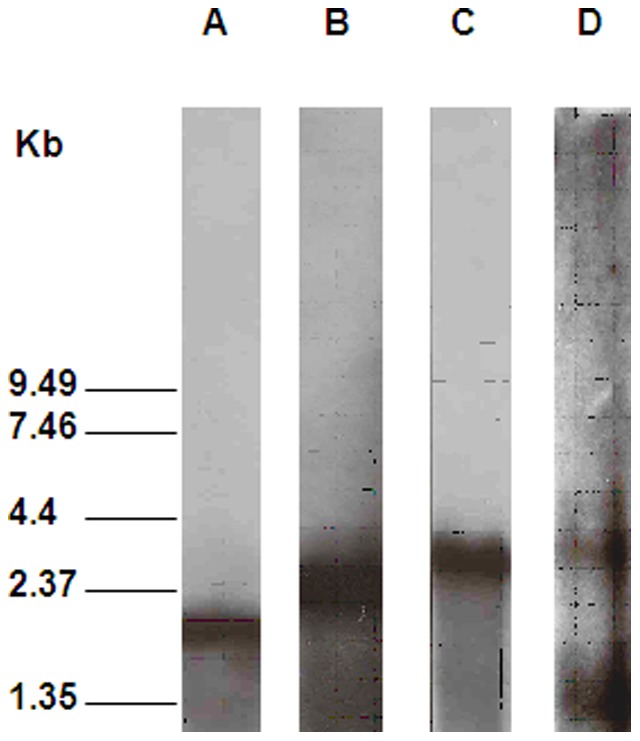
Northern blot analysis of transcripts of *cel5A* (A), *cel44O* (B), *cel5I* (C) and *cel5D* (D). Total RNAs purified from cells grown on cellulose-containing medium (ten micrograms in A, B, C and 40 micrograms in D) were separated by denaturing electrophoresis, transferred to nylon membrane and hybridized with gene-specific radioactive riboprobes.

**Figure 2:**
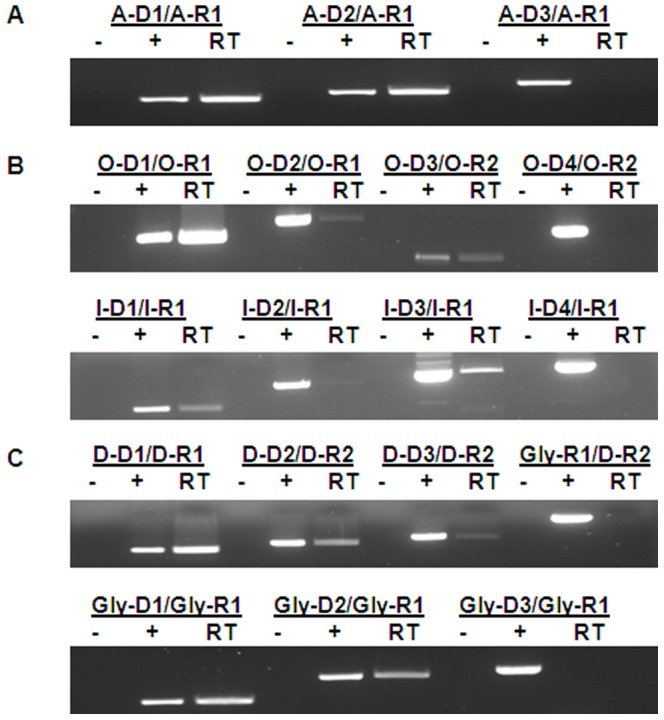
RT-PCR analysis of transcripts of *cel5A* (A), *cel44O* and *cel5I* (B) and, *cel5D* and *glyR2* (C). The cDNAs reverse transcripts obtained from total RNAs purified from cells grown in cellulose-containing medium, were amplified with the indicated pairs of primers (see Fig. 3, 4 and 5 for their locations and Table 1 for their sequences). Amplicons were analyzed by electrophoresis in 0.7% agarose gel (lanes RT). −, PCRs performed on RNAs in the absence of RT; +, PCRs performed on genomic DNA.

**Figure 3:**
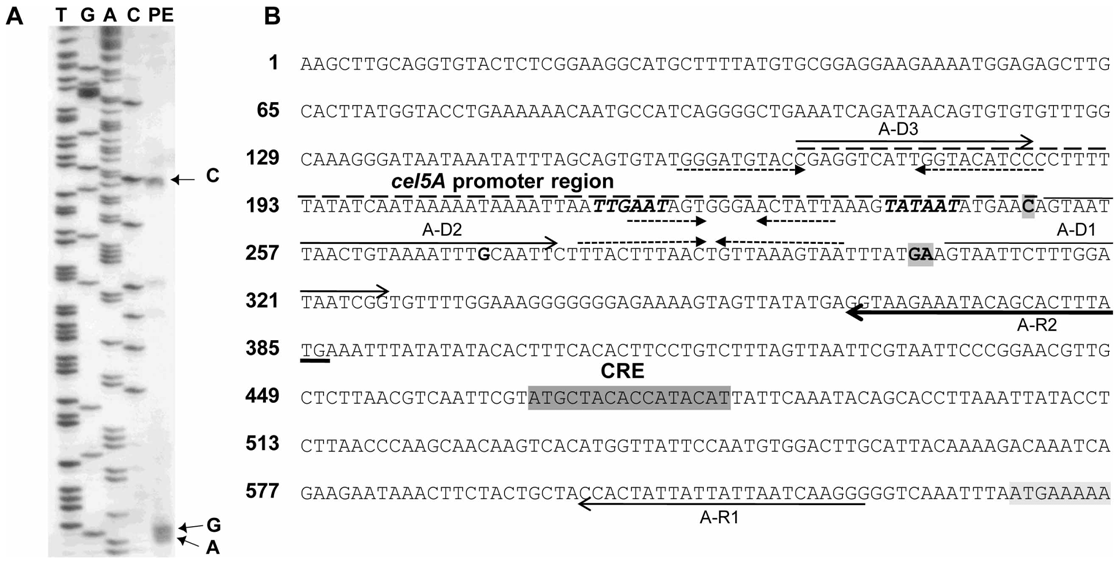
Primer extension analysis (A) and promoter region sequence (B) of *cel5A*. The A-R2 end-labeled primer was used to identify 5′ends of transcripts (lane PE in A, highlighted bold characters in B) and to generate the sequencing ladder on an 8% (wt/vol) sequencing gel. To allow direct comparison with the sequence of the strand corresponding to mRNA shown in part B, the lanes in part A are labeled with the complementary bases. The putative −35 and −10 sequences are indicated in bold italics, the putative promoter region is overlined (-----); the ORF and the putative CRE element are highlighted; the primers used for RT-PCR analysis (Fig. 2) are indicated by arrows, and inverted repeats by convergent arrows. Leader sequence starts at the +1 position of each gene and ends just before the start codon of the coding region.

*cel5A* was found to be transcribed as a monocistronic transcription unit of approximately 1.8 kb (Fig. 1A). The length of the mRNA is consistent with the length of the *cel5A* ORF (1425 bases) located upstream a putative Rho independent transcription termination site [23]. The location of the transcription initiation site was investigated using three different methods with independent mRNA preparations from cells grown on cellulose containing medium. A RT-PCR analysis showed that it is located upstream position −381/ATG (Fig. 2A, Fig. 3B). A primer extension analysis, carried out using the radiolabeled primers A-R1 and A-R2 (Fig. 3A) showed that three products were obtained with A-R2, whereas no short elongation product was obtained with A-R1 (data not shown). This analysis revealed two main 5′ ends: one might correspond to a start located at −383/ATG (Fig. 3B), which is consistent with the result obtained by RT-PCR analysis, and the second is located at −327/−328/ATG. Two faint bands corresponding to minor mRNA species were observed. It can be noticed that these 5′ends are located upstream and downstream of a putative secondary structure which might interfere with the extension procedure. The longest mRNAs was detected by RACE (GeneRacer kit, Invitrogen), in addition to several shorter mRNAs corresponding to the mRNA species revealed by primer extension (data not shown).

### *cel44O* and *cel5I* transcription

**Figure 4:**
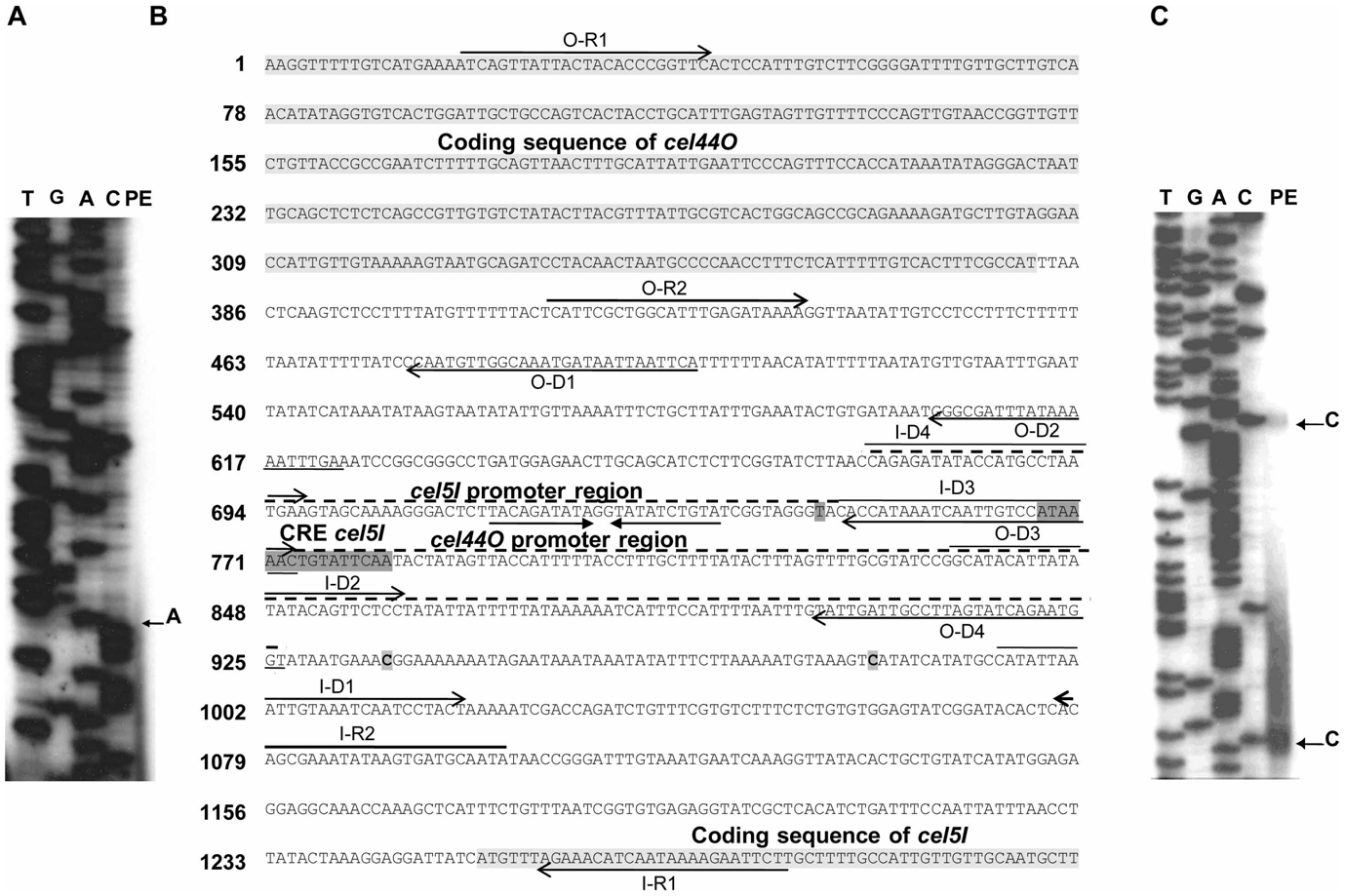
Primer extension analyses of *cel44O* and *cel5I* (A and C) and the intergenic sequence (B). The O-R2 and I-R2 end-labeled primers were used to identify 5′ends of transcripts (lane PE in A and C, respectively, highlighted bold characters in B) and to generate the sequencing ladders on an 8% (wt/vol) sequencing gel. The DNA strand shown in B corresponds to the mRNA strand of *cel5I*. The first base found at the 5′-end extremity of *cel44O* mRNA being an A (see part A), it corresponds to a bold highlighted T in the sequence shown in B. ORFs, putative promoter regions, CRE element, primers, leader sequences and inverted repeats are indicated as in Fig. 3.

*cel44O* and *cel5I* are adjacent divergent genes. Their ORFs (2586 and 2793 bases, respectively) are separated by 871 bp. They are both transcribed as monocistronic transcription units of 2.7 and 2.9 kb-long mRNAs, respectively (Fig. 1B and C) which end with a putative Rho-independent terminator 51 and 63 bases after the stop codon, respectively. The location of their transcription initiation sites was investigated using RT-PCR (Fig. 2B). As shown in figure 4B, the *cel44O* transcription initiation site is located upstream from the OD-3 and downstream from the OD-4 primers positions (−392 and −545/*cel44O* ATG, respectively). The *cel5I* transcription initiation site is located in the same area of the intergenic region upstream from position −505 and downstream from position −579/*cel5I* ATG. Low quantities of amplicons were obtained using RT-PCR on the 5′ extremity of the messengers. This result highly suggests that there are lower quantities of the 5′end-long messengers than of shorter ones, which might be explained by either the presence of two promoters, an *in vivo* processing event, or *in vitro* degradation of the mRNAs. The 5′-end extremities, detected using primer extension experiments on another set of mRNAs (Fig. 4A and C), are close to the promoter region located by RT-PCR in the case of *cel44O* whereas they are distant in the case of *cel5I*.

### *cel5D* transcription and regulation

**Figure 5:**
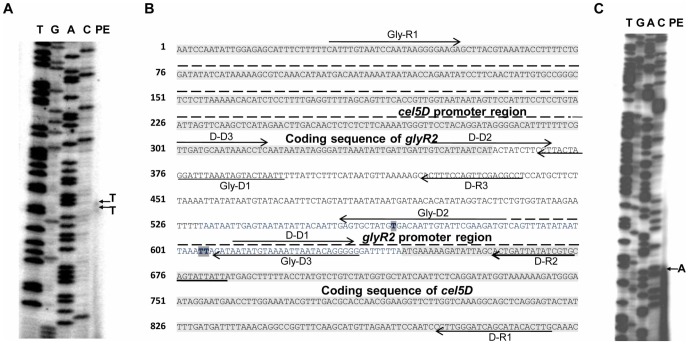
Primer extension analyses of *cel5D* (A) and *glyR2* (C) and the intergenic sequence between *glyR2* and *cel5D* (B). The D-R2 and Gly-R2 end-labeled primers were used to identify 5′ends of transcripts of *cel5D* (A) and *glyR2* (C), respectively (lane PE in A and C, highlighted bases in B) and to generate the sequencing ladder on a 8% (wt/vol) sequencing gel. The DNA strand shown in B corresponds to the mRNA strand of *cel5D*. The first base found at the 5′-end extremity of *glyR2* mRNA being an A (see part C), it corresponds to a bold highlighted T in the sequence shown in B. The lanes in parts A and C, ORF, putative promoter regions, primers, leader sequences and inverted repeats are indicated as in Fig. 3.

Several *cel5D* messengers were detected on Northern blot (Fig. 1D). The largest one of around 3 kb-long might carry *cel5D* (ORF of 1.75 kb) and the two genes located downstream from *cel5D*: a transferase (ORF of 0.33 kb) and an integrase gene (ORF of 0.66 kb). A Rho independent terminator with a low stability (ΔG = −11.2 kcal/mol, calculated with the program RNAfold) was found at 56 bases after the *cel5D* stop codon and might stop the transcription in a fraction of transcripts producing a shorter transcript (Fig. 1D). The transcription initiation site was localized using RT-PCR (Fig. 2C) upstream from position −344 and downstream from position −614/*cel5D* ATG. Primer extension experiments detected 5′-ends far away from the promoter region (Fig. 5A and B). These truncated mRNAs were also detected using RACE (data not shown).

### GlyR2 acts as an activator of *cel5D*

**Figure 6:**
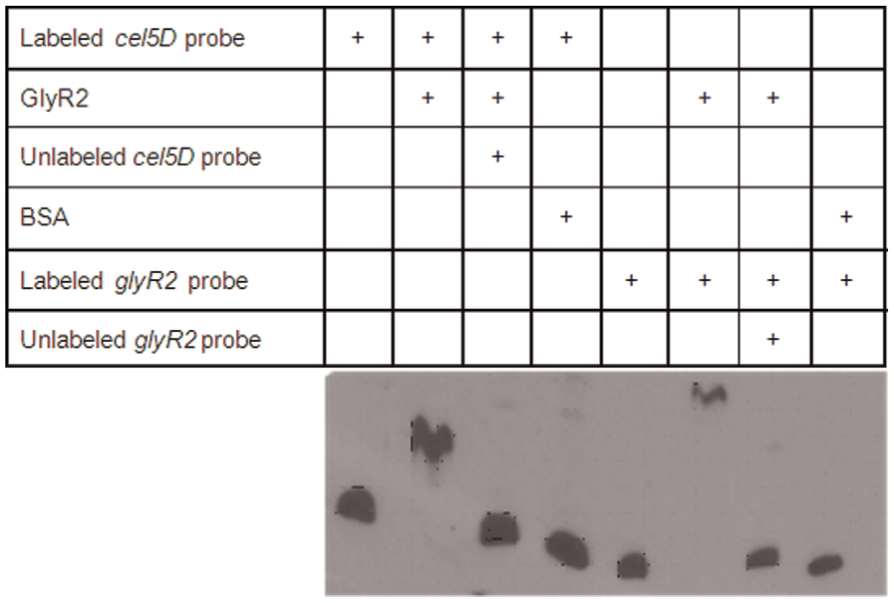
Binding of GlyR2 to the *cel5D* and *glyR2* promoter regions analysed by EMSA. Reaction mixtures contained 30 fmoles (8 ng) of a biotin-labeled 411-bp *cel5*D probe or 25 fmoles (6.9 ng) of a biotin-labeled 425-bp *glyR2* probe. Around 2 µg (57 pmoles) of GlyR2, 1 mg of BSA, 800 ng of unlabeled *cel5D* DNA probe or 690 ng of unlabeled *glyR2* DNA probe was added in certain reaction mixtures.

A gene, *glyR2*, encoding a transcriptional regulator belonging to the AraC/XylS family was found upstream from the cellulosomal cellulase *cel5D* gene in the opposite direction [18]. The AraC/XylS family of transcription regulators is one of the most common positive regulators [24], [25]. Members of the family have been categorized into three main common regulatory functions: carbon metabolism, stress response and pathogenesis. The 300 amino-acid GlyR2 polypeptide is predicted to be composed of an N-terminal binding module and a C-terminal 96 amino-acid AraC-type Helix-Turn-Helix DNA binding module. To study the function of GlyR2 and test its ability to regulate *cel5D* expression, we over-produced a C-terminal His-tagged protein in *E. coli* and partially purified it on Ni-NTA chromatography gel. Affinity of the recombinant protein to the *cel5D* and *glyR2* promoter regions was tested using electrophoretic mobility shift assay (EMSA) (Fig. 6). The promoter region of *glyR2* was located upstream from the +1 of transcription localized at −261/ATG using RT-PCR and primer extension (Figs. 2C, 5B and 5C). Two 3′-end biotin-labeled 411- and 425-bp *Pcel5D* and *PglyR2* probes, respectively were prepared by PCR using primer pairs described in Table 1 and used for the EMSA. When GlyR2 was added to the mixture before running electrophoresis, a gel-shift of the P*cel5D* and of the P*glyR2* probes was observed (Fig. 6). No gel-shift was observed when GlyR2 was replaced by BSA, indicating that the effect is protein specific. The unlabeled probe at 100-fold concentration efficiently competed for binding to GlyR2 with the biotin-labeled corresponding probe. In contrast, an unrelated DNA probe failed to compete (data not shown), which indicated that GlyR2 binds specifically to the *cel5D* and to its own gene promoter regions.

**Figure 7:**
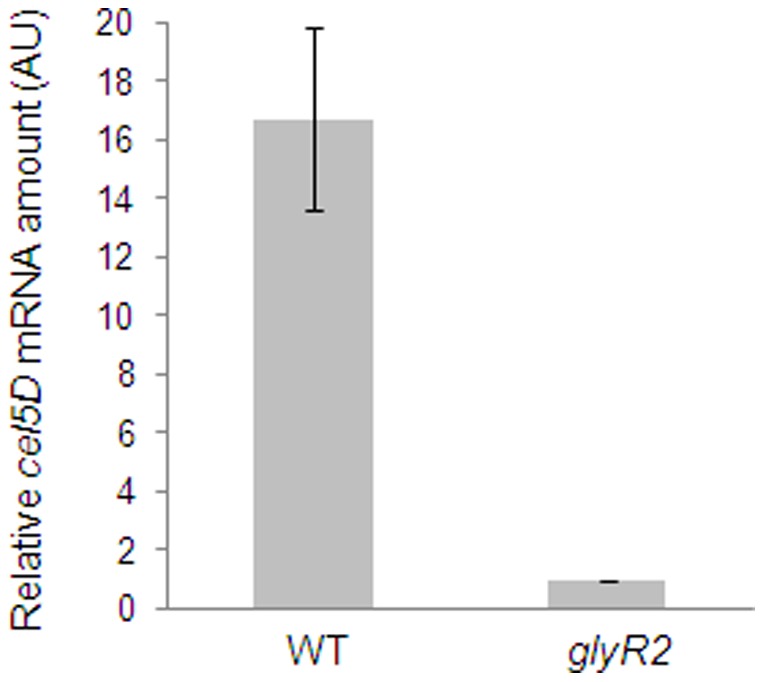
Regulation of the expression of *cel5D* by GlyR2. Wild-type (WT) and *glyR2* strains were cultured in 5 g.L^−1^ cellulose-containing MM up to mid-exponential phase. The relative RNA amount (Arbitrary Unit) of *cel5D* in the two strains was determined by qRT-PCR. cDNAs from three biological independent experiments were used and the qRT-PCR assay was performed in duplicate on each sample. The *rpoD* gene target was used as an internal control. The lowest value was normalized to 1. Mean and standard deviation are presented in the graph.

To determine whether *in vivo*, GlyR2 serves as a transcription regulator of *cel5D*, we constructed an insertional mutant of the *glyR2* gene using the ClosTron method [19]. *cel5D* messengers were analyzed from the WT strain and the *glyR2* mutant, using quantitative RT-PCR on total RNAs purified from cellulose-grown cells. As shown in figure 7, in the absence of GlyR2, the level of transcription of *cel5D* is about 16 times lower than in the WT strain. This suggests that GlyR2 acts as a transcriptional activator of *cel5D*.

### Regulation by the carbon source

**Figure 8:**
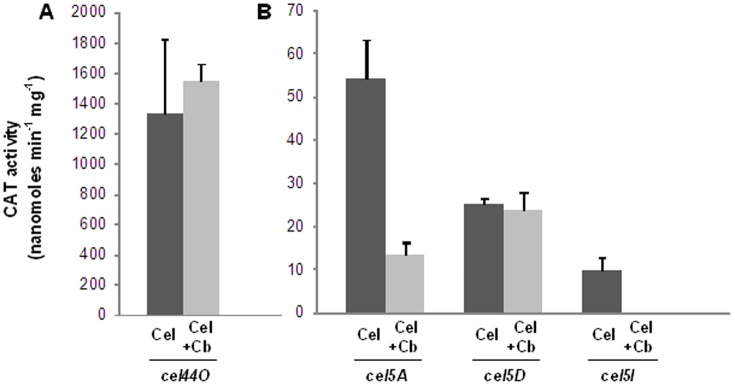
Regulation of the expression of *cel44O* (A), *cel5A*, *cel5I* and *cel5D* (B) by the carbon source. The CAT activities measured in soluble extracts of cells collected in the mid-exponential growth phase reflect the expression driven by the indicated promoters. Strains WT(pPSVPcelA), WT(pPSVPcelD), WT(pPSVcelI) and WT(pPSVcelO) were grown in 5 g.L^−1^ cellulose-containing MM (dark grey bars, Cel) or 5 g.L^−1^ cellulose+4 g.L^−1^ cellobiose-containing MM (light grey bars, Cel+Cb). The bars indicate the means of three experiments, and the error bars indicate the standard deviations.

The *cip-cel* operon was shown to be regulated by carbon catabolite repression [14]. To investigate if the expression of the studied individual genes would be sensitive to the carbon sources, we constructed transcriptional fusions of their promoter regions with the *catP* gene (see construction details in the Material and Methods section). Corresponding promoter reporter plasmids were introduced into the *C. cellulolyticum* WT strain. Soluble cell-extracts of the transformants grown on cellulose, or cellulose plus cellobiose-containing MM up to mid exponential phase were assayed for CAT activity (Fig. 8). The transcription of *cel5A* and *cel5I* was found to be sensitive to the available soluble carbon source. With the transcriptional fusion used for *cel5D*, no effect of cellobiose was observed. The activity of *cel44O* promoter was found much higher than those of the other genes whatever the available carbon source(s). Nevertheless, as *cel5D*, it does not appear to be regulated by carbon catabolite repression.

## Discussion

The goal of this work was to investigate the expression and regulation of various individual *cel* genes, namely *cel5A*, *cel44O*, *cel5I* and *cel5D*. Conversely to the *cip-cel* operon, these genes are transcribed as monocistronic units of transcription, except *cel5D*. It might be hypothesized that the transcription unit of the latter gene was disturbed by the insertion of a mobile element at its 3′ end; indeed the two genes co-transcribed with *cel5D* are predicted to encode an integrase gene and a transferase gene. As for the *cip-cel* operon, a large leader sequence was found in the 5′ end of messengers except for *cel5A* and *cel44O*. The leader sequences of *cel5I* and *cel5D* are assumed to be subjected to processing events as suggested by the results of the primer elongations and RT-PCR experiments. Such phenomenon has already been observed at the 5′-end of the *cip-cel* operon transcripts [13], [14].

*cel5A* and *cel5I* transcription levels are lower when a soluble carbon source is available *cel5A* and *cel5I* appear to be regulated by a carbon catabolite repression mechanism. One putative CRE sequence was found in the area of the *cel5A* promoter based on sequence comparison with the *Bacillus subtilis* consensus (WTGNNARCGNWWWCAW, [26]), This sequence shares 13 bases with the consensus (Fig. 3B). Four CRE sequences sharing also 13 bases with the *B. subtilis* consensus were found downstream from the promoter region of *cel5I* (data not shown). One of them shares also 12 bases with the consensus sequence of *Clostridium difficile* (RRGAAAANGTTTTCWW, [27])(Fig. 4B). The location of the CRE sequences found in the leader sequences of *cel5A* and *cel5I* is consistent with a road-block mechanism of transcription inhibition by a repressor located at the CRE site. Such a promoter-downstream location of a functional CRE sequence has been identified to regulate the *cip-cel* operon of *C. cellulolyticum*. It would be interesting to mutate these sequences and test the carbon catabolite repression sensitivity of these modified promoter regions. Putative CRE sequences were also found in the leader region of *cel5D* and *cel44O* (data not shown) but no catabolite repression could be observed using transcriptional fusions. These sequences may be dysfunctional. Further investigations would be necessary to prove it.

One regulator gene (*glyR2*) was found upstream from *cel5D*, and the recombinant corresponding regulator was found to specifically interact with the *cel5D* promoter region and with the promoter of its own gene. In *C. thermocellum*, GlyR3 was the first transcriptional regulator of glycoside hydrolase genes identified. It binds specifically to a near perfect 18-bp palindrome in the *celC* promoter region and acts as a repressor in *in vitro* transcription assay [28]. No similar palindromic sequences could be found in the promoter regions of *cel5D* and *glyR2*. Footprint experiments would precisely identify the GlyR2 binding sites. GlyR2 belongs to the AraC/XylS family which mainly comprises activators. In *glyR2* mutant cells grown in cellulose-containing medium, the *cel5D* transcription level was found much lower than in the control strain, indicating that GlyR2 acts as an activator. AraC/XylS regulators are known to be activated by an inducer [25]. The nature of its inducer is not yet known.

Regulation of the expression of genes encoding the cellulolytic/hemicellulolytic system of *Clostridium cellulolyticum* appears to involve various mechanisms. Four different mechanisms have already been described: carbon catabolite repression regulates the expression of the *cip-cel* operon, *cel5A* and *cel5I*, mRNA processing coupled to secondary messengers differential stability contributes to fine tune the expression of individual genes of the *cip-cel* operon [13], a two component system might be involved in the regulation of the *xyl-doc* cluster [29], and finally an activator of the AraC/XylS family was demonstrated to regulate the expression of *cel5D* in this study.
